# Annotations

**Published:** 1909-02-13

**Authors:** 


### Annotations.

### Lumbar Anæsthesia.

The time seems to have arrived when some systematic endeavour should be made to get at the entire truth about the advantages and disadvantages of lumbar anæsthesia. From time to time occasional fatalities are reported; but no impartial census of their exact frequency, of the risks of the proceeding in fact, seems to have been published. We make these remarks more especially in view of a fatality at Wandsworth due to stovaine inected into the *theca spinalis* for the purpose of procuring anæsthesia. On the one hand we see several surgeons using the method freely and reporting good results; on the other a good number of fatalities reported. It seems to be unfortunate and unnecessary, but at the same time absolutely true, that professional anæsthetists prefer the older methods of securing general anæsthesia, without being able to refute the advocates of the lumbar method from first-hand experience. It is quite probable that their distrust is justifiable, for there seems to be a general idea among medical men who have watched some of the surgeons who have worked most with the method in London that its dangers have been considerably minimised or underestimated in their contributions to literature. And yet as long as the surgeons are able to taunt the anæsthetists with unwillingness to try the new plan, it must be very difficult to get a just estimate of its exact risks. If a full dress debate could be arranged by the Anæsthetic Section of the Royal Society of Medicine, to which surgeons of eminence could be invited, and the whole matter thoroughly discussed, a great service to medicine, surgery, the profession, and the public would be rendered.

### New Type for "The Times."

The premier daily newspaper of the Empire, whatever the criticisms which may have been raised at various times against its policy or its methods, has always preserved a high reputation for the excellent quality of its news, of its paper, and of its printing. From a typographical point of view, indeed, the *Times* has long been an admirable example to the world of daily journalism, and has earned the thanks of all serious newspaper readers who could afford to consider their eyesight, as well as their tastes in politics, literature and affairs, in the selection of a morning paper. Surprise was therefore mingled with our sense of gratified approval when we learnt on February 5, on the authority of the *Times* itself, that in the following issue and thenceforward, that journal would increase the size and improve the quality of its smaller type. The motive for this generous action—we had almost written, for this act of super-erogation, comparable to the gilding of refined gold or the adding of another hue unto the typographical rainbow—was stated to be a desire to meet the wishes of some of its readers. The new type has been selected after careful experiments—optical, ophthalmological, and technical; and it bears the approval of two such eminent oculists as Sir Anderson Critchett and Mr. Brudenell Carter. We have compared the new type with that previously in use; and the new is without doubt a definite and obvious improvement, which will surely satisfy even those exigeant readers at whose special request the change has been made. The rapidity with which modern newspapers are printed, and the immense numbers of copies which are made from the same type, render legibility and actual comfort in reading difficult to ensure. The relatively high price of the *Times* thus gives it a great advantage over its contemporaries in these respects, since better paper can be used, and more deliberation can be exercised in the various processes of composing and printing. But, even so, we cannot withhold our congratulations upon this recent step towards perfection. Readers who are interested in the ophthalmological and hygienic aspects of legibility in printed characters should study Mr. Brudenell Carter's short commentary on the change of fount, which appears in the same issue of the *Times* as the announcement of the coming alteration.

### The Medical Register.

It is of much importance from every point of view that the official Register of the General Medical Council should be accurate and complete. A special effort is being made by the Registrar to ensure that the 1910 issue of the Medical Register may contain the names of all registered practitioners now living, and that no names of those who have died may remain upon it. With this in view a circular was posted on the first of this month to every registered practitioner, excepting only those in his Majesty's Services, at the address recorded on the Register, inquiring whether he has ceased to practise or has changed his address. The letter which we publish this week from the Registrar draws attention to the importance of every medical man replying to this inquiry at the earliest possible date, and we willingly give this further publicity to it, since it is in the interest of the whole profession, as well as of each individual practitioner, that the Register for 1910 should be perfectly accurate. Those who by any chance have failed to receive the circular should communicate at once with the Registrar; for if no answer is returned from any qualified medical man by August 1st of this year, his name is liable to be erased from the Register forthwith; and we understand that the General Medical Council, in the interests of the profession and the public, intend to exercise this right on the present occasion. If for any reason a permanent address cannot be given, some other should be supplied to which official communications may be directed. If a practitioner's name is removed from the Register under Section XIV. of the Medical Act of 1858, for failure to answer an official inquiry as to his existence and whereabouts from the Council's Registrar, it is, of course, always possible for him to have it restored on establishing his identity; but this may be a matter of some difficulty or inconvenience, and until it is done he is, for the time being, not a registered medical practitioner, and can claim none of those legal privileges to which he was entitled whilst his name remained upon the *current* copy of the Medical Register.

